# The CNS Myelin Proteome: Deep Profile and Persistence After Post-mortem Delay

**DOI:** 10.3389/fncel.2020.00239

**Published:** 2020-08-19

**Authors:** Olaf Jahn, Sophie B. Siems, Kathrin Kusch, Dörte Hesse, Ramona B. Jung, Thomas Liepold, Marina Uecker, Ting Sun, Hauke B. Werner

**Affiliations:** ^1^Proteomics Group, Max Planck Institute of Experimental Medicine, Göttingen, Germany; ^2^Department of Neurogenetics, Max Planck Institute of Experimental Medicine, Göttingen, Germany

**Keywords:** oligodendrocyte, myelin proteome, central nervous system (CNS), demyelination, post-mortem delay, autopsy, label-free proteomics, transcriptome

## Abstract

Myelin membranes are dominated by lipids while the complexity of their protein composition has long been considered to be low. However, numerous additional myelin proteins have been identified since. Here we revisit the proteome of myelin biochemically purified from the brains of healthy c56Bl/6N-mice utilizing complementary proteomic approaches for deep qualitative and quantitative coverage. By gel-free, label-free mass spectrometry, the most abundant myelin proteins PLP, MBP, CNP, and MOG constitute 38, 30, 5, and 1% of the total myelin protein, respectively. The relative abundance of myelin proteins displays a dynamic range of over four orders of magnitude, implying that PLP and MBP have overshadowed less abundant myelin constituents in initial gel-based approaches. By comparisons with published datasets we evaluate to which degree the CNS myelin proteome correlates with the mRNA and protein abundance profiles of myelin and oligodendrocytes. Notably, the myelin proteome displays only minor changes if assessed after a post-mortem delay of 6 h. These data provide the most comprehensive proteome resource of CNS myelin so far and a basis for addressing proteomic heterogeneity of myelin in mouse models and human patients with white matter disorders.

## Introduction

In the central nervous system (CNS) of vertebrates, the velocity of nerve conduction is accelerated by the insulation of axons with multiple layers of myelin membrane provided by oligodendrocytes (Nave and Werner, 2014; Snaidero and Simons, 2017). Compared to other cellular membranes myelin is unusually enriched for lipids, in particular cholesterol, galactolipids and plasmalogens (Norton and Poduslo, 1973a; Schmitt et al., 2015; Poitelon et al., 2020). Indeed, the biogenesis of myelin may involve the coalescence of lipid-rich membrane-microdomains in the oligodendroglial secretory pathway (Lee, 2001; Chrast et al., 2011). Notably, the dominant CNS myelin protein, proteolipid protein (PLP), displays a high affinity to cholesterol-rich membrane-microdomains (Simons et al., 2000; Werner et al., 2013). PLP and other cholesterol-associated myelin proteins may thus enhance the coalescence and intracellular traffic of prospective myelin membranes (Schardt et al., 2009). Indeed, both cholesterol and PLP are rate-limiting for myelination, as demonstrated by the dysmyelination observed in mice lacking oligodendroglial cholesterol synthesis (Saher et al., 2005) or PLP-expression (Yool et al., 2001; Möbius et al., 2008; de Monasterio-Schrader et al., 2013).

As a key stage of myelination, the compaction of adjacent CNS myelin layers requires myelin basic protein (MBP), as evidenced by the complete lack of compact myelin in the CNS of MBP-deficient *shiverer*-mice (Roach et al., 1985). It is now thought that MBP both displaces filamentous actin and cytoskeleton-associated proteins (Nawaz et al., 2015; Zuchero et al., 2015; Snaidero et al., 2017) and saturates negative charges of the headgroups of phosphatidylinositol-4,5-bisphosphate (PIP_2_) on the cytoplasmic myelin membrane surfaces (Musse et al., 2008; Nawaz et al., 2009, 2013) thereby pulling together and compacting myelin membranes at the major dense line (Raasakka et al., 2017).

It has been noted already in the early 1970s that PLP and MBP constitute the most abundant CNS myelin proteins. At that time the methods were developed for the enrichment of myelin from nervous tissue by sucrose density gradient centrifugation (Norton and Poduslo, 1973b; Erwig et al., 2019a) separation by one-dimensional (1D)-polyacrylamide gel electrophoresis (SDS-PAGE) and protein staining using Buffalo Black (Morris et al., 1971) Fast Green (Morell et al., 1972) or Coomassie Blue (Magno-Sumbilla and Campagnoni, 1977). Indeed, only few bands were visible that we now know are mainly constituted by PLP, MBP and cyclic nucleotide phosphodiesterase (CNP; Sprinkle et al., 1983). Deficiency of CNP in mice impairs both the ultrastructure of myelin and the long-term preservation of axonal integrity (Lappe-Siefke et al., 2003; Edgar et al., 2009; Patzig et al., 2016; Snaidero et al., 2017).

Evidently, the protein composition of myelin is more complex when considering that various additional myelin proteins have been identified, including myelin associated glycoprotein (MAG; Quarles, 2007; Myllykoski et al., 2018), myelin oligodendrocyte glycoprotein (MOG; Johns and Bernard, 1999; von Büdingen et al., 2015), and claudin 11 (CLDN11; Gow et al., 1999; Denninger et al., 2015). This insight motivated attempts to utilize the emerging mass spectrometric techniques to approach all myelin proteins at once, thereby covering the entire myelin proteome. Indeed, purified myelin is suited for systematic assessment of its molecular constituents (De Monasterio-Schrader et al., 2012; Gopalakrishnan et al., 2013). Most early approaches involved 2D-gels (Taylor et al., 2004; Vanrobaeys et al., 2005; Werner et al., 2007), soon to be complemented by gel-free shotgun-approaches (Vanrobaeys et al., 2005; Roth et al., 2006; Dhaunchak et al., 2010) and hybrid workflows (Ishii et al., 2009). However, first systematic information on the relative abundance of myelin proteins was achieved by label-free quantification involving peptide-separation by liquid chromatography (LC) coupled to detection with quadrupole time-of-flight (QTOF) mass spectrometery (MS) (Jahn et al., 2009) or by chemical peptide labeling with isobaric tags for relative and absolute quantitation (iTRAQ) and subsequent LC-MS-analysis (Manrique-Hoyos et al., 2012). A meta-analysis of the approaches to the myelin proteome published by 2012 is available (De Monasterio-Schrader et al., 2012). Since then, label-free protein quantification by LC-MS has proven useful in the differential analysis of myelin in mouse models including mice lacking PLP, CNP, or MAG (Patzig et al., 2016). For example, this approach allowed identifying cytoskeletal septin filaments to stabilize the ultrastructure of CNS myelin, thereby preventing the formation of pathological myelin outfoldings (Patzig et al., 2016; Erwig et al., 2019b).

The intention of this work was to both establish an updated comprehensive compendium of the proteins associated with CNS myelin and to accurately quantify their relative abundance, as recently achieved for the proteome of myelin in the peripheral nervous system (Siems et al., 2020). To this aim we combined various gel-based and gel-free proteomic techniques. In particular, we used nano-flow ultra-performance liquid chromatography (nanoUPLC) for peptide separation and an ion mobility-enabled QTOF-system for label-free protein quantification by data-independent acquisition (DIA) mass spectrometry in an alternating low and elevated energy mode (MS^E^). While the MS^E^-mode allows quantifying myelin proteins with the required dynamic range of over four orders of magnitude, an ion mobility-enhanced version thereof [referred to as ultra-definition (UD)-MS^E^] covers about twice as many myelin-associated proteins, though at the expense of dynamic range. Our workflow thus facilitates both to reliably quantify the exceptionally abundant PLP, MBP, and CNP and to appreciate the complexity of low-abundant myelin constituents.

## Materials and Methods

### Animals

Male c57BL/6N wild-type mice at postnatal day 75 (P75) were used for all experiments except for the differential analysis of myelin purified from brains immediately frozen after dissection compared to a post-mortem delay of 6 h at room temperature (Figure 4), for which female c57BL/6N wild-type mice at P56 were used. Mice were bred and kept in the animal facility of the Max Planck Institute of Experimental Medicine and sacrificed by cervical dislocation. For the procedure of sacrificing mice for subsequent preparation of tissue, all regulations given in the German animal protection law (TierSchG §4) are followed. Since sacrificing of rodents is not an experiment on animals according to §7 Abs. 2 Satz 3 TierSchG, no specific authorization or notification is required for the present work.

### Myelin Purification

A myelin-enriched light-weight membrane fraction was biochemically purified from mouse brains by sucrose density centrifugation and osmotic shocks as recently described in detail (Erwig et al., 2019a). Mice were sacrificed by cervical dislocation at the indicated ages as three biological replicates per condition (*n* = 3). Protein concentration was determined using the DC Protein Assay Kit (Bio-Rad). Initial quality control by gel electrophoresis and silver staining of gels was performed as described (de Monasterio-Schrader et al., 2013; Joseph et al., 2019). Briefly, samples were separated on a 12% SDS-PAGE gel (1 h at 200 V) using the Bio-Rad system, fixated overnight in 10% [v/v] acetic acid and 40% [v/v] ethanol and then washed in 30% ethanol (2 × 20 min) and ddH_2_O (1 × 20 min). For sensitization, gels were incubated 1 min in 0.012% [v/v] Na_2_S_2_O_3_ and subsequently washed with ddH_2_O (3 × 20 s). For silver staining, gels were impregnated for 20 min in 0.2% [w/v] AgNO_3_/0.04% formaldehyde, washed with ddH_2_O (3 × 20 s) and developed in 3% [w/v] Na_2_CO_3_/0.04% [w/v] formaldehyde. The reaction was stopped by exchanging the solution with 5% [v/v] acetic acid.

### Gel-Based Proteome Analysis of Myelin

Gel-electrophoretic separation of myelin proteins with different pre-cast gel systems (Serva) was performed essentially as recently described in detail (Erwig et al., 2019a). Briefly, 1D separations were performed with 5 μg protein load before (pre-wash) or after (post-wash) subjecting myelin to consecutive high-salt and high-pH washing/centrifugation cycles as previously described (Werner et al., 2007; Jahn et al., 2013). Automated tryptic in-gel digestion of proteins in gel bands (Schmidt et al., 2013) and protein identification by LC-MS was performed as described (Ott et al., 2015). For 2D separations, myelin was first delipidated by methanol/chloroform precipitation and 300 μg protein was loaded on a 24 cm immobilized non-linear pH-gradient 3-12 strip (Serva) by active rehydration (Erwig et al., 2019a). Automated tryptic in-gel digestion of proteins in gel spots and protein identification by MALDI-TOF mass spectrometry was performed as described (Jahn et al., 2006; Werner et al., 2007).

### Label-Free Quantification of Myelin Proteins

In-solution digestion of myelin proteins according to an automated filter-aided sample preparation (FASP) protocol (Erwig et al., 2019a) and LC-MS-analysis by different MS^E^-type data-independent acquisition (DIA) mass spectrometry approaches was performed as recently established for PNS myelin (Siems et al., 2020). Briefly, protein fractions corresponding to 10 μg myelin protein were dissolved in lysis buffer (1% ASB-14, 7 M urea, 2 M thiourea, 10 mM DTT, 0.1 M Tris pH 8.5) and processed according to a CHAPS-based FASP protocol in centrifugal filter units (30 kDa MWCO, Merck Millipore). After removal of the detergents, protein alkylation with iodoacetamide, and buffer exchange to digestion buffer [50 mM ammonium bicarbonate (ABC), 10% acetonitrile], proteins were digested overnight at 37°C with 400 ng trypsin. Tryptic peptides were recovered by centrifugation and extracted with 40 μl of 50 mM ABC and 40 μl of 1% trifluoroacetic acid (TFA), respectively. Combined flow-through were directly subjected to LC-MS-analysis. For quantification according to the TOP3 approach (Silva et al., 2006), aliquots were spiked with 10 fmol/μl of yeast enolase-1 tryptic digest or Hi3 EColi standard (Waters Corporation), the latter containing a set of quantified synthetic peptides derived from *E. coli*. Chaperone protein ClpB.

Nanoscale reversed-phase UPLC separation of tryptic peptides was performed with a nanoAcquity UPLC system equipped with a Symmetry C18 5 μm, 180 μm × 20 mm trap column and a HSS T3 C18 1.8 μm, 75 μm × 250 mm analytical column (Waters Corporation) maintained at 45°C. Peptides were separated over 120 min at a flow rate of 300 nl/min with a gradient comprising two linear steps of 3–35% mobile phase B (acetonitrile containing 0.1% formic acid) in 105 min and 35–60% mobile phase B in 15 min, respectively. Mass spectrometric analysis of tryptic peptides was performed using a Synapt G2-S QTOF mass spectrometer equipped with ion mobility option (Waters Corporation). UDMS^E^ analysis was performed in the ion mobility-enhanced data-independent acquisition mode with drift time-specific collision energies as described in detail (Distler et al., 2014a, 2016). Continuum LC-MS data were processed using Waters ProteinLynx Global Server (PLGS) and searched against a custom database compiled by adding the sequence information for yeast enolase 1, *E. coli* Chaperone protein ClpB and porcine trypsin to the UniProtKB/Swiss-Prot mouse proteome and by appending the reversed sequence of each entry to enable the determination of false discovery rate (FDR). Precursor and fragment ion mass tolerances were automatically determined by PLGS and were typically below 5 ppm for precursor ions and below 10 ppm (root mean square) for fragment ions. Carbamidomethylation of cysteine was specified as fixed and oxidation of methionine as variable modification. One missed trypsin cleavage was allowed. Minimal ion matching requirements were two fragments per peptide, five fragments per protein, and one peptide per protein. The FDR for protein identification was set to 1% threshold.

For post-identification analysis including TOP3 quantification of proteins, the freely available software ISOQuant^1^ was used (Kuharev et al., 2015). Only peptides with a minimum length of seven amino acids that were identified with scores above or equal to 5.5 in at least two runs were considered. FDR for both peptides and proteins was set to 1% threshold and only proteins reported by at least two peptides (one of which unique) were quantified as parts per million (ppm) abundance values (i.e., the relative amount (w/w) of each protein in respect to the sum over all detected proteins). The Bioconductor R packages “limma” and “*q*-value” were used to detect significant changes in protein abundance by moderated t-statistics as described (Ambrozkiewicz et al., 2018; Siems et al., 2020). For proteome profiling of wild-type myelin by MS^E^ and UDMS^E^, three independent experiments were performed, each with three biological replicates and sample processing with duplicate digestion and injection, resulting in a total of 12 LC-MS runs per experiment. Abundance values in ppm are given as averages of the four technical replicates per biological replicate and only proteins quantified in at least two out of three experiments are reported in the proteome resource (Supplementary Table S1). Proteins identified as contaminants from blood (albumin, hemoglobin) or hair cells (keratins) were removed from the list. Proteome profiling comparing wild-type myelin without and with post-mortem delay (Figure 4 and Supplementary Table S2) was performed with three biological replicates and duplicate digestion, resulting in a total of 6 LC-MS runs per condition. Data acquisition was performed in the DRE-UDMS^E^ mode (Siems et al., 2020) i.e., a deflection device was cycled between full (100% for 0.4 s) and reduced (5% for 0.4 s) ion transmission during one 0.8 s full scan, thereby providing a compromise between identification rates and dynamic range.

### Interpretation of Single-Cell Resolution Transcriptome Data

Published single-cell RNA-sequencing (scRNA-seq) gene expression matrices from datasets GSE60361 (Zeisel et al., 2015), GSE75330 (Marques et al., 2016), and GSE113973 (Falcão et al., 2018), were obtained from Gene Expression Omnibus (GEO) and analyzed using R package Seurat v3.1.0 (Butler et al., 2018; Stuart et al., 2019). Mature oligodendrocyte cell populations were selected from each dataset as specified in the results section and normalized gene counts were used for calculating average expression profiles across single cells. Bulk proteome and transcriptome datasets specified in the results section were used as supplied in the Supplementary Tables to the respective publications.

### Deposition, Visualization and Analysis of Proteomic Data

The mass spectrometry proteomics data have been deposited to the ProteomeXchange Consortium via the PRIDE (Perez-Riverol et al., 2019) partner repository with dataset identifier PXD020007. Pie chart, scatter plots, volcano plot and heatmap were prepared in Microsoft Excel 2013 and GraphPad Prism 8. Area-proportional Venn diagrams were prepared using BioVenn (Hulsen et al., 2008)^2^. Trans-membrane domains were predicted using TMHMM Server v. 2.0 (Krogh et al., 2001)^3^ and Phobius (Käll et al., 2007)^4^.

## Results

### Proteome Analysis of CNS Myelin

**FIGURE 1:**
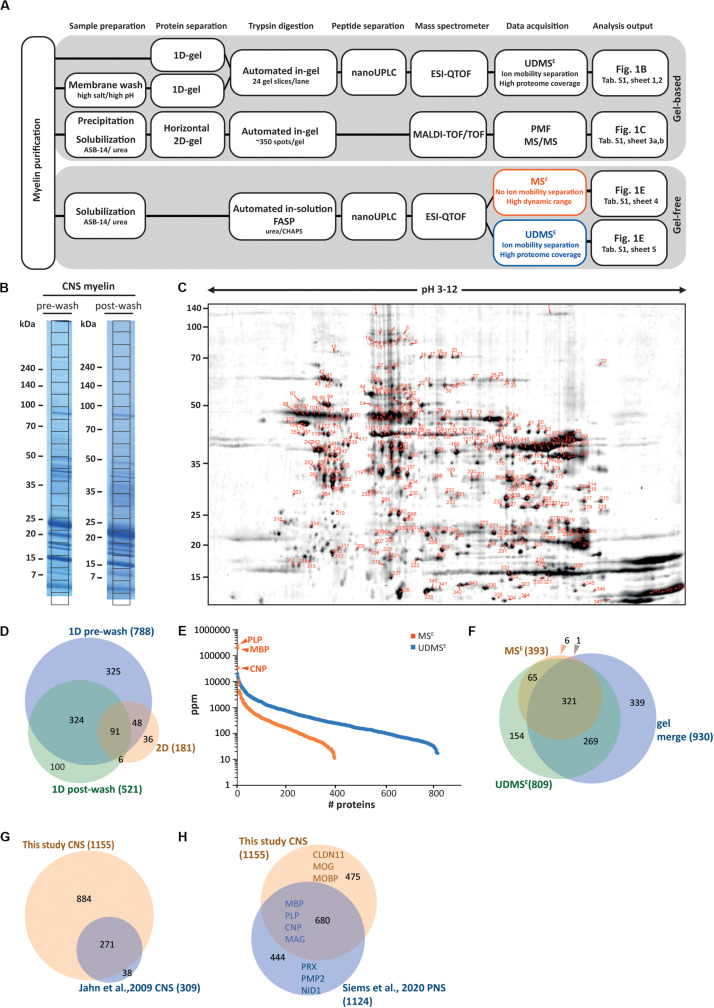
Proteome analysis of CNS myelin. **(A)** Schematic illustration of the gel-based (top) and gel-free (bottom) proteomic workflow to approach CNS myelin purified from the brains of wild-type c57Bl/6N mice dissected at P75. Note that gel-free proteome analysis enables largely automated sample processing and omits labor-intense gel-electrophoresis, thus reducing hands-on time. **(B)** One-dimensional gel-separation of CNS myelin. Myelin was separated by SDS-PAGE without (pre-wash) or upon (post-wash) depleting soluble and peripheral membrane proteins by an additional step of high-pH and high-salt conditions. Proteins were visualized with colloidal Coomassie (CBB250). The denoted grid subdivides each lane into 24 equally sized slices, which were excised for automated tryptic digest, peptide separation by nanoUPLC and data acquisition using an ESI-QTOF mass spectrometer, thereby identifying 788 (pre-wash) and 521 (post-wash) proteins, respectively (see Supplementary Table S1). **(C)** Two-dimensional gel-separation of CNS myelin. Myelin was two-dimensionally separated using a 2D-IEF/SDS-PAGE with isoelectric focusing (IEF) in a 24 cm gel strip with nonlinear pH-gradient (pH 3–12) as the first and 10–15% acrylamide gradient SDS-PAGE (25.5 × 20 cm, gel thickness 0.65 mm) as the second dimension. Proteins were visualized by colloidal Coomassie staining; protein spots were excised, subjected to automated tryptic in-gel digestion and MALDI-TOF mass spectrometry, thereby identifying 181 non-redundant proteins from 352 spots (Supplementary Table S1). **(D)** Venn diagram comparing the number of proteins identified in CNS myelin by the three gel-based approaches. **(E)** Number and relative abundance of proteins identified in myelin purified from the brains of wild-type mice using two gel-free data acquisition modes (MS^E^, UDMS^E^). Note that MS^E^ (orange) identifies comparatively fewer proteins in purified myelin but provides a dynamic range of more than four orders of magnitude. UDMS^E^ (blue) identifies a larger number of proteins but provides a dynamic range of only about three orders of magnitude. Note that the dynamic range of MS^E^ is required for the quantification of the exceptionally abundant myelin proteins proteolipid protein (PLP), myelin basic protein (MBP) and cyclic nucleotide phosphodiesterase (CNP). Samples were analyzed in three biological replicates with four technical replicates each (duplicate digestion and injection). For datasets see Supplementary Table S1. ppm, parts per million. **(F)** Venn diagram comparing the number of proteins identified in CNS myelin by MS^E^, UDMS^E^ and gel-based approaches. **(G)** Venn diagram of the proteins identified in CNS myelin in this study compared with those identified in a previous approach (Jahn et al., 2009). **(H)** Venn diagram comparing the proteins identified in CNS myelin in this study with those previously identified in PNS myelin (Siems et al., 2020). Selected marker proteins are denoted.

To purify CNS myelin, we applied an established protocol (Erwig et al., 2019a) to prepare a light-weight membrane fraction from the brains of healthy c57Bl/6N-mice at P75. Aiming to systematically identify myelin-associated proteins we used five complementary approaches as summarized in Figure 1A. As the most straightforward way of preparing myelin for proteomic analysis, we separated proteins by 1D-SDS-PAGE and sectioned the lane into 24 equally sized slices (Figure 1B), which we subjected to automated tryptic in-gel digest followed by LC-MS-analysis, thereby identifying 788 proteins (Supplementary Table S1). When we subjected myelin to an additional washing step of high-pH and high-salt conditions (Figure 1B) to deplete soluble and peripheral membrane proteins before 1D-SDS-PAGE-separation and mass spectrometry, we identified 521 proteins (Supplementary Table S1). To establish a reference map of myelin proteins including proteoforms, we increased the resolving power of protein separation by subjecting myelin to 2D-gel electrophoresis with isoelectric focusing (IEF) in the first and horizontal SDS-PAGE in the second dimension (Figure 1C). We stained the comprised proteins with colloidal Coomassie (CBB250), picked CBB250-labeled gel-plugs (i.e., protein spots) for automated tryptic in-gel digestion and identified the proteins by peptide mass fingerprint (PMF) and MS/MS-fragment ion mass spectra, both acquired on a MALDI-TOF mass spectrometer. We identified 181 non-redundant proteins from 352 spots (Supplementary Table S1). Thereby we expanded our previous myelin protein map (131 non-redundant proteins from 217 spots, Werner et al., 2007), mainly owing to increased resolution in the first dimension by utilizing longer IEF-strips with a wider pH-range (Erwig et al., 2019a). When comparing the proteins identified using the three gel-based approaches we found a total of 930 proteins with a fair overlap (Figure 1D).

Considering that contemporary gel-free, label-free proteomic approaches allow the simultaneous identification and quantification of proteins (Neilson et al., 2011; Distler et al., 2014b) we subjected myelin to a workflow of solubilization using ASB-14 and high-urea conditions, automated tryptic in-solution digest by filter-aided sample preparation (FASP), fractionation of peptides by nanoUPLC, and ESI-QTOF mass spectrometry. This workflow was recently established for peripheral myelin (Siems et al., 2020). Importantly, the utilized data-independent acquisition (DIA)-strategy with data acquisition in the MS^E^-mode allows the simultaneous quantification and identification of all peptides entering the mass spectrometer, and thereby, when signal intensities are correlated with a spike protein of known concentration (TOP3 method; Silva et al., 2006; Ahrné et al., 2013) the reliable quantification of proteins based on peptide intensities. When subjecting myelin to LC-MS-analysis using MS^E^ we quantified 393 proteins (Supplementary Table S1; labeled in orange in Figure 1E) with a false discovery rate (FDR) of <1% and an average sequence coverage of 38.6%. Notably, MS^E^ quantitatively covered myelin proteins with a dynamic range of over four orders of magnitude parts per million (ppm), thereby allowing quantification of the exceptionally abundant PLP and MBP. When using the ultra-definition (UD)-MS^E^-mode, in which the ion mobility option provides an orthogonal dimension of peptide separation after liquid chromatography and before mass measurement, we identified and quantified 809 proteins (Supplementary Table S1; labeled in blue in Figure 1E) with an average sequence coverage of 35.0%. UDMS^E^ thus identified about twice as many proteins as MS^E^. However, the larger number of proteins identified by UDMS^E^ went along with a compressed dynamic range of about three orders of magnitude ppm, which is insufficient to reliably quantify the most abundant myelin constituents including PLP, MBP, and CNP. The data acquisition mode-dependent differences in both numbers of quantified proteins and dynamic range are best explained by UDMS^E^ achieving more efficient precursor-fragment ion alignment and precursor fragmentation upon ion mobility separation of peptides (Distler et al., 2014a, 2016) which causes a ceiling effect for the detection of exceptionally intense peptide signals and thus a compressed dynamic range as previously observed for PNS myelin (Siems et al., 2020).

When comparing the proteins identified by MS^E^, UDMS^E^ and gel-based approaches we found a reasonably high overlap (Figure 1F). Comparison of the 1155 proteins identified in CNS myelin in the present study with those 309 identified >10 years ago with the methodological standards of that time (Jahn et al., 2009) shows a remarkably high overlap as well as an about three-fold increase in the number of identified proteins (Figure 1G). Notwithstanding that a number of the identified proteins will originate from other cellular sources that contaminate purified myelin, we believe that many of them are indeed low-abundant constituents of the non-compact compartments of myelin.

A comparison of the proteins identified in CNS myelin with the recently established PNS myelin proteome (Siems et al., 2020) confirms that numerous proteins are present in both, but also that many proteins were identified exclusively in either CNS or PNS myelin (Figure 1H). Together, the evolving technical standards of in-solution sample preparation and MS^E^-type DIA mass spectrometry allows to comprehensively identify and quantify proteins in myelin. However, only MS^E^ (but not UDMS^E^) provides a dynamic range suited to address the relative abundance of the exceptionally abundant PLP, MBP, and CNP. As importantly, the evolution of gel-free methods shifts the major workload in myelin proteome analysis from manual sample handling to data analysis, with much less hands-on time required when compared to gel-based approaches.

### Relative Abundance of CNS Myelin Proteins

**FIGURE 2:**
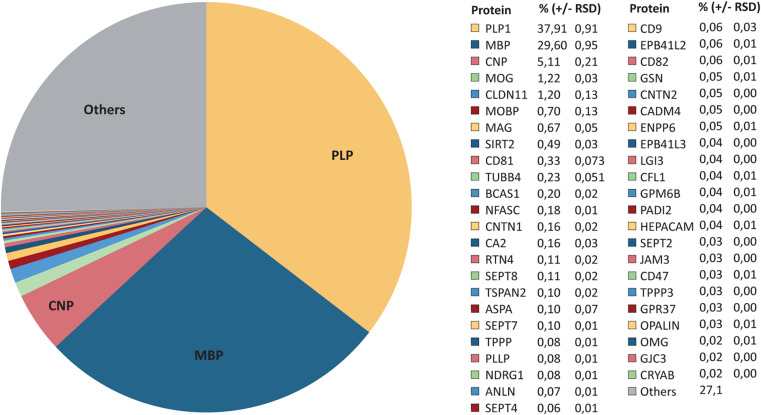
Relative abundance of CNS myelin proteins. Pie chart of the MS^E^ dataset shown in Figure 1E and Supplementary Table S1. The relative abundance of known myelin proteins is given as percent with relative standard deviation (% ±RSD). Note that known myelin proteins constitute approximately 73% of the total myelin protein; proteins so far not independently validated as myelin proteins constitute about 27%.

As MS^E^ provides the best possible dynamic range (Figure 1E), we evaluated the relative abundance of all 393 proteins identified in myelin by MS^E^ (Figure 2 and Supplementary Table S1). As per this dataset PLP constitutes 38% of the total myelin protein [±1% relative standard deviation (RSD)]. MBP, CNP, and MOG constitute 30% (±1%), 5% (±0.2%), and 1% (±0.03%) of the total myelin protein, respectively (Figure 2). However, the present assessment of CNS myelin by MS^E^ extends well beyond the most abundant myelin constituents, thus quantifying known myelin proteins including the tetraspan-proteins CLDN11, CD81, TSPAN2, PLLP, CD9, CD82, GPM6B, and GJC3, the immunoglobulin-domain containing cell-surface proteins MAG, NFASC, CNTN1, RTN4, CNTN2, CADM4, HEPACAM, JAM3, CD47, and OMG, the enzymes SIRT2, CA2, and ASPA, the cytoskeletal and cytoskeleton-associated proteins TUBB4, SEPT2, SEPT4, SEPT7, SEPT8, TPPP, ANLN, GSN, CFL1, and PADI2 as well as MOBP, BCAS1, NDRG1, opalin, and CRYAB (Figure 2). By MS^E^, 46 known myelin proteins account for approximately 80% of the total myelin protein (Figure 2). The remaining 27% is constituted by 347 proteins not yet validated as myelin constituents by independent methods.

### Comparison to Related Datasets

**FIGURE 3:**
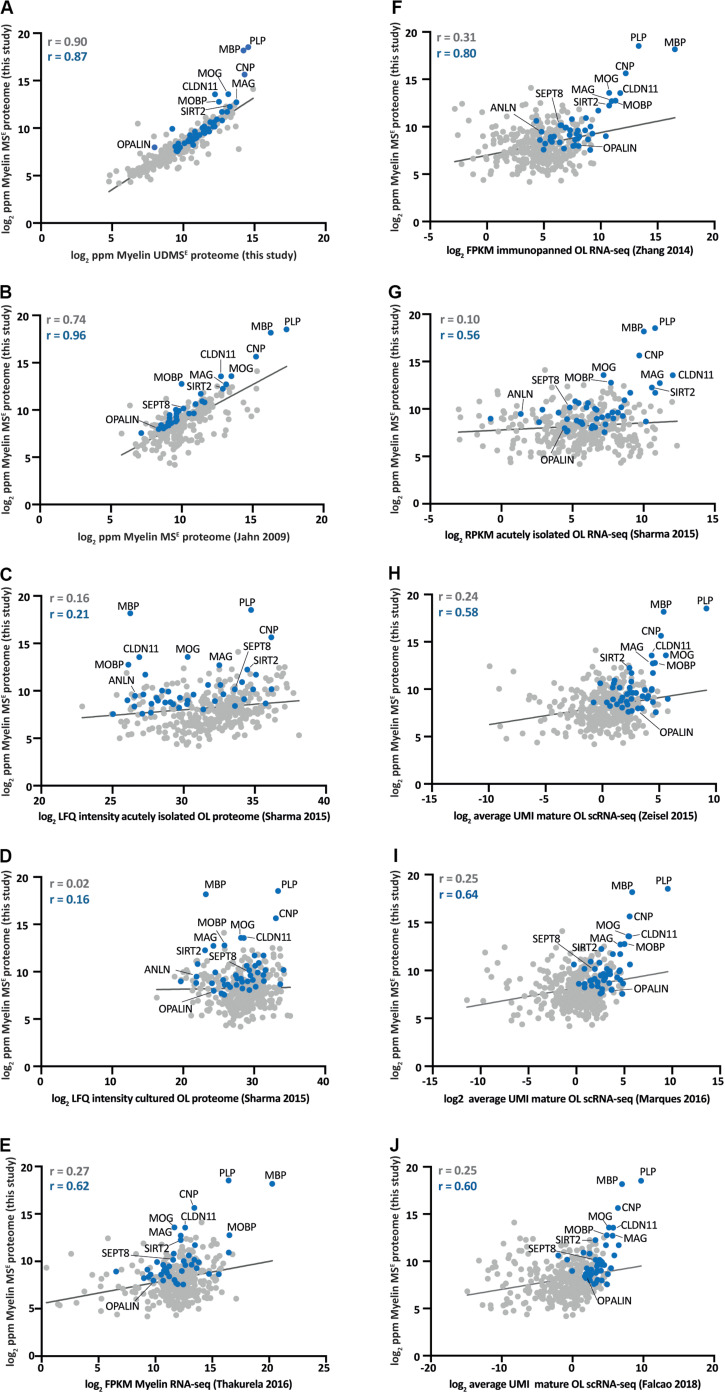
Comparison of the myelin proteome with proteome and transcriptome profiles of myelin and oligodendrocytes. **(A)** Log_2_-transformed relative abundance of the proteins identified in myelin in this study by MS^E^ plotted against their log_2_-transformed relative abundance as quantified by UDMS^E^. Data points representing known myelin proteins as specified in Figure 2 are labeled in blue; all other data points in gray. The correlation coefficient (*r*) was calculated for all proteins identified by MS^E^ (displayed in gray) and specifically for the known myelin proteins (given in blue). The regression line is plotted for orientation. ppm, parts per million. **(B)** Same as **(A)** but plotted against the myelin proteome as previously assessed by MS^E^ (Jahn et al., 2009). **(C)** Same as **(A)** but plotted against the proteome of acutely isolated O4-immunopositive oligodendrocytes (Sharma et al., 2015). LFQ, label-free quantification. **(D)** Same as **(A)** but plotted against the proteome of O1-immunopositive primary oligodendrocytes cultured for 4 days *in vitro* (DIV) (Sharma et al., 2015). **(E)** Same as **(A)** but plotted against the RNA-seq-based transcriptome of myelin purified from the brains of mice (Thakurela et al., 2016). FPKM, fragments per kilobase of exon model per million reads mapped. **(F)** Same as **(A)** but plotted against the RNA-seq-based transcriptome of oligodendrocytes immunopanned using MOG-specific antibodies (Zhang et al., 2014). **(G)** Same as **(A)** but plotted against the RNA-seq-based transcriptome of acutely isolated O4-immunopositive oligodendrocytes (Sharma et al., 2015). RPKM, reads per kilobase per million mapped reads. **(H)** Same as **(A)** but plotted against the scRNA-seq-based transcriptome of mature oligodendrocytes in the mouse cortex and hippocampus [mean of all 484 cells in clusters Oligo5 and Oligo6 in Zeisel et al. (2015)]. UMI, unique molecular identifiers. **(I)** Same as **(A)** but plotted against the scRNA-seq-based transcriptome of mature oligodendrocytes in 10 regions of the mouse CNS [mean of all 2748 cells in clusters OL1 – OL6 in Marques et al. (2016)]. **(J)** Same as **(A)** but plotted against the scRNA-seq-based transcriptome of mature oligodendrocytes in the mouse spinal cord [mean of all 617 cells in clusters MOL2-Ct and MOL5/6-Ct in Falcão et al. (2018)].

An increasing number of studies provides mRNA or protein abundance profiles of myelin or oligodendrocytes. To systematically compare the CNS myelin proteome with these profiles, we correlated our MS^E^-dataset (Figure 2 and Supplementary Table S1) via the gene name entries with related datasets for which quantitative information is publicly available (Figure 3).

We first plotted the present MS^E^ and UDMS^E^-datasets (Supplementary Table S1) against each other (Figure 3A). Considering that the same starting material has been assessed it is not unexpected that the datasets correlate well, as reflected by a correlation coefficient of 0.90 (Figure 3A). Most visibly diverging from the linear regression line are the most abundant myelin proteins PLP, MBP, CNP, MOG, and CLDN11, reflecting that the dynamic range of UDMS^E^ is compressed in the high ppm-range compared to that of MS^E^ (also see Figure 1E). We then compared the present MS^E^-dataset with an independent myelin proteome dataset previously established by MS^E^ (Jahn et al., 2009). We calculated a somewhat lower correlation coefficient of 0.74 (Figure 3B), probably owing to the previous use of a predecessor mass spectrometer generation that provided a considerably lower dynamic range. Yet, in conjunction with the high overlap between the proteins identified in the present and the previous study (Jahn et al., 2009) (Figure 1G), myelin proteome analysis emerges as fairly robust across independently purified starting material and different generations of mass spectrometers. We next compared the MS^E^-dataset to the proteome of acutely isolated O4-immunopositive oligodendrocytes (Figure 3C) as determined by label-free quantification (LFQ) using data-dependent acquisition (DDA) on an orbitrap mass spectrometer and MaxQuant-software (Sharma et al., 2015). The O4-antibody preferentially immunolabels oligodendrocytes at the progenitor (OPC) and pre-myelinating stages (Sommer and Schachner, 1981; Bansal et al., 1989; Goldman and Kuypers, 2015); the correlation coefficient was calculated as 0.16 (Figure 3C). A correlation coefficient of 0.02 (Figure 3D) was found when comparing the MS^E^-dataset with the LFQ-intensity profile of O1-immunopositive primary oligodendrocytes after 4 days *in vitro* (DIV) (Sharma et al., 2015). The myelin proteome as determined here is thus more closely related to the proteome of acutely isolated O4-immunopositive oligodendrocytes than to that of O1-immunopositive primary oligodendrocytes 4 DIV.

We then compared the MS^E^-dataset with various available mRNA-abundance profiles. When comparing the MS^E^-dataset to the transcriptome of purified CNS myelin as determined by RNA-seq (Thakurela et al., 2016) we calculated a correlation coefficient of 0.27 (Figure 3E). Interestingly, the comparison between the MS^E^-dataset and the RNA-seq-based transcriptome of oligodendrocytes immunopanned from the cortex using antibodies against MOG (Zhang et al., 2014) revealed a roughly comparable correlation coefficient of 0.31 (Figure 3F). Notably, MOG-immunopositivity labels myelinating oligodendrocytes, implying that the stage of oligodendrocyte differentiation must be considered when judging dataset correlations. It is thus not surprising that a somewhat lower correlation coefficient of 0.10 (Figure 3G) was calculated when comparing the MS^E^-dataset with the RNA-seq-based transcriptome of acutely isolated O4^+^- oligodendrocytes (Sharma et al., 2015). Finally, we compared the MS^E^-dataset to several scRNA-seq-based transcriptome datasets (Zeisel et al., 2015; Marques et al., 2016; Falcão et al., 2018). To this aim we calculated the mean transcript abundance as average count reads per unique molecular identifier (UMI) of the cells in those clusters that reflect mature oligodendrocytes. When comparing the MS^E^-dataset to mature oligodendrocytes sorted from the mouse cortex and hippocampus [all 484 cells in clusters Oligo5 and Oligo6 in Zeisel et al. (2015)], we find a correlation coefficient of 0.24 (Figure 3H). Importantly, we find a roughly similar correlation coefficient when comparing the MS^E^-dataset to mature oligodendrocytes sorted from 10 regions of the mouse CNS [all 2748 cells in clusters MOL1–MOL6 in Marques et al. (2016)] (Figure 3I) or to mature oligodendrocytes sorted from the spinal cord of mice [all 617 cells in clusters MOL2-Ct and MOL5/6-Ct in Falcão et al. (2018)] (Figure 3J).

Together, when judging correlations between large datasets evaluating mRNA and protein abundance profiles of oligodendrocytes and myelin, aspects to be considered include the method of sample preparation, the stage of oligodendrocyte differentiation and the methodology of analysis. Yet, roughly similar correlation coefficients were calculated when comparing the myelin proteome with various proteomic and transcriptomic approaches to the molecular profiles of oligodendrocytes.

### Persistence of the Myelin Proteome Upon Post-mortem Delay

**FIGURE 4:**
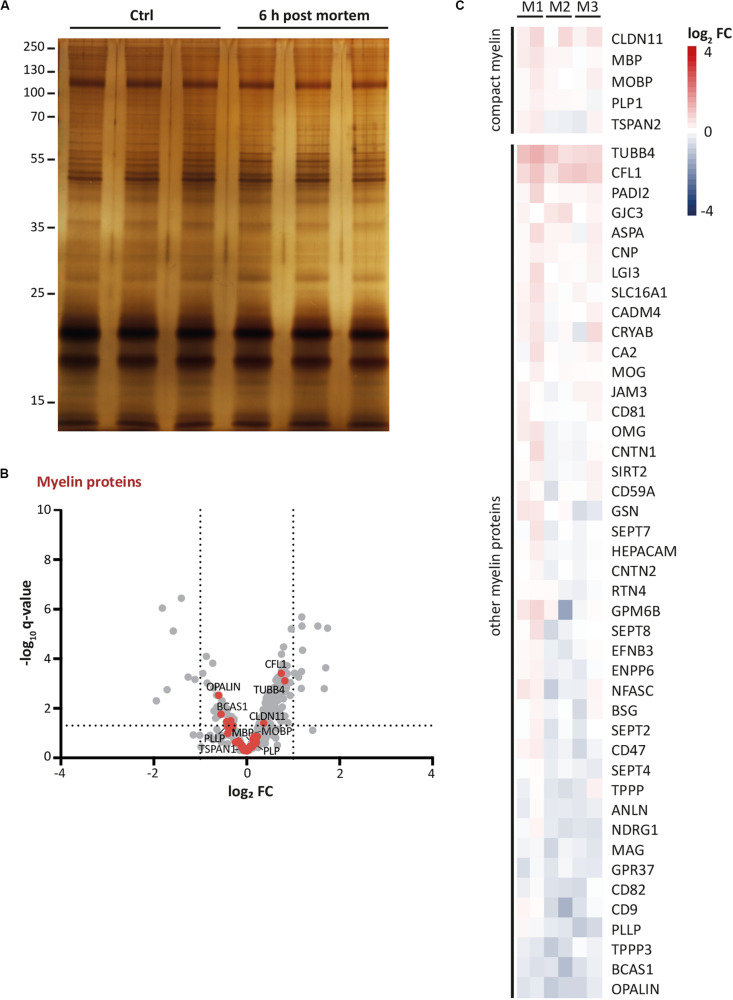
Persistence of myelin proteins upon post-mortem delay. **(A)** Myelin purified from the brains of mice at P56 was separated by SDS-PAGE (0.5 μg protein load) and proteins were visualized by silver staining. Myelin of brains frozen upon a post-mortem delay of 6 h at room temperature was compared with myelin of brains frozen immediately upon dissection (Ctrl). Note the similar band pattern. Gel shows *n* = 3 biological replicates per condition. **(B)** Volcano plot representing differential proteome analysis by DRE-UDMS^E^ to compare myelin purified from brains upon post-mortem delay with myelin of brains immediately frozen upon dissection. For entire dataset see Supplementary Table S1. Data points represent proteins quantified in myelin purified from mouse brains frozen after a post-mortem delay of 6 h at room temperature compared to immediately frozen brains and are plotted as the log_2_-transformed fold-change (FC) on the *x*-axis against the –log_10_-transformed *q*-value on the *y*-axis. Vertical stippled lines mark a 2-fold/0.5-fold change (FC) as significance threshold. Horizontal stippled line represents a –log_10_-transformed *q*-value of 1.301, reflecting a *q*-value of 0.05 as significance threshold. Data points highlighted in red represent known myelin proteins as specified in Figure 4C. Note that no known myelin protein exceeds the fold-change significance threshold. **(C)** Heatmap displaying known myelin proteins as highlighted by the red data points in Figure 4B. Heatmap shows reduced (blue) or increased (red) abundance in myelin purified from brains after post-mortem delay. Each horizontal line corresponds to the fold-change (FC) of a distinct myelin protein compared to its average abundance in control myelin plotted on a log_2_ color scale. Heatmap displays 6 replicates, i.e., three biological replicates per condition (M1, M2, M3) with two technical replicates each.

Autopsy material from human patients and healthy-appearing controls is increasingly evaluated by systematic molecular profiling, as exemplified by the recent snRNA-seq-based assessment of oligodendroglial transcriptional profiles in multiple sclerosis patients (Jäkel et al., 2019). Notably, the use of autopsy material involves a post-mortem delay between the death of a subject and the collection of a biopsy. However, post-mortem delay may affect sample integrity and thus data validity. Considering that proteomic analysis of myelin in mice is usually performed upon freezing of samples immediately after dissection, we asked whether the myelin proteome can also be assessed upon post-mortem delay. We thus purified myelin from the brains of c57Bl/6N-mice to compare the myelin proteome between mice after a post-mortem delay of 6 h at room temperature with that of mice upon sample freezing immediately after dissection. Upon SDS-PAGE-separation and silver staining, no signs of major degradation were evident and the band patterns appeared essentially similar (Figure 4A). We next subjected myelin to routine differential proteome profiling by UDMS^E^ with dynamic range enhancement (DRE-UDMS^E^) (Supplementary Table S2). Using this data acquisition mode with intermediate features as to identification rates and dynamic range (for methodological details see Siems et al., 2020) we found that known myelin proteins displayed only minor differences as visualized in a volcano plot (red data points in Figure 4B) and a heatmap (Figure 4C). Indeed, no known myelin protein exceeded the threshold of a log_2_-fold transformed fold-change (FC) of −1/+1, i.e., a 2-fold increased or 0.5-fold diminished relative abundance. Together, the myelin proteome displays only minor changes upon a post-mortem delay of 6 h, implying that proteomic assessment of myelin purified from autopsy samples appears feasible.

## Discussion

Understanding the molecular complexity of the nervous system involves molecular profiling of cells and cellular specializations including myelin. Here we combined various proteomic approaches for comprehensive coverage of the CNS myelin proteome and identified 1155 proteins in myelin biochemically purified from the brains of mice. We note that gel-based methods involving separation at the protein level facilitated a slightly higher identification rate compared to gel-free methods comprising *in situ*-digestion of the entire proteome, likely because of the pre-fractionation effect inherent to the former. On the other hand, gel-free data acquisition by UDMS^E^ also enabled deep qualitative coverage while necessitating considerably less input material and manual sample handling.

Importantly, the MS^E^-data acquisition mode covered a dynamic range of over four orders of magnitude of protein abundance. Indeed, compared to a previous approach (Jahn et al., 2009) the technical advancements implemented in the current mass spectrometer generation now allow reliable quantification of myelin proteins spanning from the exceptionally abundant PLP and MBP to low-abundant constituents including oligodendrocyte myelin glycoprotein (OMG) (Wang et al., 2002), oligodendrocytic myelin paranodal and inner loop protein (OPALIN) (Golan et al., 2008; Kippert et al., 2008) and the G-protein coupled receptor GPR37 (Yang et al., 2016). For PLP, MBP and CNP our quantification is in accordance with but specifies prior estimates based on 1D-gel separation and various protein staining techniques, in which they were proposed to constitute 30–45%, 22–35%, and 4–15% of the total myelin protein, respectively (Morell et al., 1972, 1973; Banik and Smith, 1977; Deber and Reynolds, 1991). Notably, it also shifts our previous MS^E^-based estimates for PLP and MBP (Jahn et al., 2009) toward higher relative abundance, with the lower dynamic range of the mass spectrometers at that time being the most likely reason for the former under-quantification. It is not surprising that PLP, MBP, and CNP have overshadowed less abundant myelin constituents in initial gel-based approaches when considering the exceptional dynamic range of the relative abundance of myelin proteins. Together, the myelin proteome provided here provides an updated comprehensive compendium and re-adjusts the relative abundance of CNS myelin proteins.

Do true myelin proteins exist that escape proteomic identification? As exemplified by myelin and lymphocyte protein (MAL) (Schaeren-Wiemers et al., 2004), the tryptic digest of some myelin proteins may result in peptides incompatible with mass spectrometric detection; their identification would require the use of proteases other than trypsin. We also note that some low-abundant signaling proteins with potent functions in regulating myelination may be assumed to localize to myelin *in vivo* but were not mass spectrometrically identified, as exemplified by the G-protein coupled receptors GPR17 (Chen et al., 2009) and GPR56/ADGRG1 (Ackerman et al., 2015; Giera et al., 2015) and the Ig-domain containing LINGO1 (Mi et al., 2005). It is currently speculative if these proteins are preferentially expressed in oligodendroglial cell bodies rather than myelin membranes or during the stages of oligodendrocyte differentiation that precede myelination. It is also speculative if enhanced mass spectrometric sensitivity would facilitate their identification in myelin. Indeed, we can not formally exclude that these proteins may be identified if less rigorous criteria were applied (e.g., demanding only one peptide per protein), which may be sufficient for identification but not for the reliable quantification of proteins as aimed at in the present study. Importantly, however, lower stringency may not only identify more true myelin constituents but also false-positive hits. This is a concern, in particular when considering that the myelin-enriched fraction may comprise up to 5% contaminants from other cellular sources (De Monasterio-Schrader et al., 2012). We note that currently no biochemical method is available that allows preventing this limitation. Yet, comparing various datasets yields systematic information, for example on the presence of a transcript in oligodendrocytes as expected for a CNS myelin protein.

Mutations affecting genes that encode classical myelin proteins including PLP, CNP, MAG, TUBB4, and ASPA cause severe neurological disorders including hypomyelinating leukodystrophies (HLD) and spastic paraplegias (SPG) (Kaul et al., 1993; Saugier-Veber et al., 1994; Simons et al., 2013; Lossos et al., 2015; Al-Abdi et al., 2020) (Table 1). However, current sequencing efforts also identify disease-causing genes that encode less well-characterized proteins. Notably, most types of leukodystrophies and spastic paraplegias are caused by mutations affecting genes of which the transcripts are enriched in neurons, astrocytes or microglia rather than oligodendrocytes (Nave and Werner, 2014; van der Knaap et al., 2019). For newly identified disease genes, thus, evaluating mRNA-expression using transcriptome datasets and presence of the protein in myelin using the present myelin proteome resource may serve as a useful entry point into identifying the primarily affected cell type. For example, mutations of the *HSPD1* gene cause HLD4 or SPG13 (Hansen et al., 2002; Magen et al., 2008) and mutations of the *TMEM63A* gene cause HLD19 (Yan et al., 2019) (Table 1). Considering that both transcripts are expressed in oligodendrocytes as per transcriptome datasets and both proteins are comprised in the myelin proteome, the disease mechanisms may involve primary impairment of the biogenesis, maintenance or functions of myelin.

**TABLE 1:** Comparison of proteins identified in CNS myelin and disease genes associated with white matter pathology.

Protein name	Gene symbol	OMIM#	Gene Locus	Disease
Aldehyde dehydrogenase 3a2	*ALDH3A2*	609523	17p11.2	Sjogren-Larsson Syndrome
Aspartoacylase	*ASPA*	608034	17p13.2	Canavan disease
Atlastin GTPase 1	*ATL1*	606439	14q22.1	SPG 3A
Cathepsin *D*	*CTSD*	610127	11p15.5	Ceroid lipofuscinosis
Contactin-associated protein 1	*CNTNAP1*	602346	17q21.2	LCCS 7
Cyclic nucleotide phosphodiesterase	*CNP*	123830	17q21.2	HLD
Dynamin 2	*DNM2*	602378	19p13.2	LCCS 5
Endoplasmic reticulum lipid raft-associated protein 2	*ERLIN2*	611605	8p11.23	SPG 18
Glial fibrillary acidic protein	*GFAP*	137780	17q21.31	Alexander disease
Glutamate-Ammonia ligase	*GLUL*	138290	1q25.3	Glutamine-deficiency, congenital
Heat-shock 60-kD protein 1	*HSPD1*	118190	2q33.1	HLD 4, SPG 13
Hepatocyte cell adhesion molecule	*HEPACAM*	611642	11q24.2	MLC 2
Junctional adhesion molecule 3	*JAM3*	613730	11q25	HDBSCC
Magnesium transporter NIPA1	*NIPA1*	608145	15q11.2	SPG 6
Monoacylglycerol lipase ABHD12	*ABHD12*	613599	20p11.21	PHARC
Myelin basic protein	*MBP*	159430	18q23	18q deletion syndrome
Myelin-associated glycoprotein	*MAG*	159460	19q13.12	SPG 75
Myelin-oligodendrocyte glycoprotein	*MOG*	159465	6p22.1	Narcolepsy 7
Neurofascin	*NFASC*	609145	1q32.1	NEDCPMD
Phosphoglycerate dehydrogenase	*PHGDH*	606879	1p12	PHGDH deficiency, NLS 1
Phosphoserine aminotransferase 1	*PSAT1*	610936	9q21.2	PSAT deficiency, NLS 2
Prosaposin	*PSAP*	176801	10q22.1	Metachromatic Leukodystrophy
Proteolipid protein	*PLP1*	300401	Xq22.2	Pelizaeus-Merzbacher disease, SPG 2
Transmembrane protein 63a	*TMEM63A*	618685	1q42.12	HLD 19
Tubulin beta 4a	*TUBB4A*	602662	19p13.3	Dystonia 4, HLD 6

*Proteins listed fulfill three criteria: (1) mass spectrometric identification in purified CNS myelin, (2) transcript expression in oligodendrocytes and (3) gene mutations associated with diseases involving pathology of myelin or the white matter. For some of the proteins with additional expression in astrocytes, microglia or neurons it is presently unknown whether loss/gain of function in oligodendrocytes is causative of the disease. HLD, Hypomyelinating Leukodystrophy; SPG, Spastic Paraplegia; LCCS, Lethal Congenital Contracture Syndrome; MLC, Megalencephalic leukoencephalopathy with subcortical cysts; NLS, Neu-Laxova syndrome; HDBSCC, Hemorrhagic destruction of the brain, subependymal calcification and cataracts; PHARC, polyneuropathy, hearing loss, ataxia, retinitis pigmentosa and cataract; NEDCPMD, neurodevelopmental disorder with central and peripheral motor dysfunction.*

Dysfunctions of oligodendrocytes and myelin contribute to the neuropathology in a growing number of neurodegenerative disorders and their respective mouse models, including Rett syndrome (Nguyen et al., 2013), amyotrophic lateral sclerosis (Kang et al., 2013), Down syndrome (Olmos-Serrano et al., 2016), Alzheimer’s disease (Nasrabady et al., 2018) and multiple sclerosis (Factor et al., 2020). Considering that molecular assessments now frequently involve autopsy material, it is motivating that our data imply that myelin proteome analysis appears well possible post-mortem, at least up to a 6 h delay. A systematic understanding of the abundance profiles of all myelin proteins in the healthy brain and in myelin-related disorders may contribute to comprehending myelin-related physiology and pathophysiology. Myelin proteome analysis as pursued here provides a basis for addressing possible proteomic heterogeneity of myelin in dependence of CNS region, age and species, as well as in mouse models and human patients with white matter disorders.

## Data Availability Statement

The mass spectrometry proteomics data have been deposited to the ProteomeXchange Consortium via the PRIDE (Perez-Riverol et al., 2019) partner repository with dataset identifier, PXD020007.

## Ethics Statement

Ethical review and approval was not required for the animal study because for the procedure of sacrificing mice for subsequent preparation of tissue, all regulations given in the German animal protection law (TierSchG §4) are followed. Since sacrificing of rodents is not an experiment on animals according to §7 Abs. 2 Satz 3 TierSchG, no specific authorization or notification is required for the present work.

## Author Contributions

KK, DH, RJ, TL, and MU performed the experiments. SS analyzed the data and performed the statistical analysis. TS wrote the code to interpret single cell resolution transcriptome data. OJ and HW conceived, designed, and directed the study. HW wrote the manuscript with major contributions by SS and OJ. All the authors contributed to revising the manuscript and approved the submitted version.

## Conflict of Interest

The authors declare that the research was conducted in the absence of any commercial or financial relationships that could be construed as a potential conflict of interest.

## Footnotes

1 www.isoquant.net

2 https://www.biovenn.nl/

3 www.cbs.dtu.dk/services/TMHMM

4 http://phobius.sbc.su.se/

## Supplementary Material

TABLE S1 Compendium of the mouse CNS myelin proteome. **(Sheet 1)** 1D gel separation of proteins followed by in-gel digestion and LC-MS analysis (788 proteins). **(Sheet 2)** 1D gel separation of proteins after membrane wash followed by in-gel digestion and LC-MS analysis (521 proteins). **(Sheet 3a)** 2D gel separation of proteins followed by in-gel digestion and MALDI-TOF-MS: spot annotation of the master gel shown in Figure 3C (352 gel spots). **(Sheet 3b)** 2D gel separation of proteins followed by in-gel digestion and MALDI-TOF-MS: non-redundant protein list (181 proteins). **(Sheet 4)** In-solution digestion of proteins and label-free quantification by MS^E^ (393 proteins). **(Sheet 5)** In-solution digestion of proteins and label-free quantification by UDMS^E^ (809 proteins). **(Sheet 6)** Compendium providing information on the approach by which a myelin-associated protein was identified and on predicted transmembrane domains (1155 proteins).

TABLE S2 Label-free quantification of proteins in CNS myelin purified from mouse brains without (Ctrl) and after 6 h post-mortem delay (PMD6h) by DRE-UDMS^E^. Related to Figure 4. **(Sheet 1)** Dataset.
